# Re-creation of Horsepox Virus

**DOI:** 10.1128/mSphere.00079-18

**Published:** 2018-03-07

**Authors:** Michael J. Imperiale

**Affiliations:** a University of Michigan, Ann Arbor, Michigan, USA

**Keywords:** biosecurity, poxvirus, synthetic biology

## EDITORIAL

Today, *mSphere* is publishing two commentaries on a controversial topic: should the horsepox virus have been reconstructed? A group led by David Evans at University of Alberta was funded by the company Tonix Pharmaceuticals, Inc., in New York to build this virus as a potential step toward a new smallpox vaccine (1).

This work occurs at a time when much attention is being paid to dual use research of concern (DURC): research that is performed due to its potential benefit, but the results of which could potentially be misused for nefarious purposes. In this case, the debate focuses on the benefit of a new smallpox vaccine versus the risk that someone may unleash variola virus itself, the causative agent of smallpox, on a largely unvaccinated human population. There is no argument against the fact that the eradication of smallpox was one of the greatest achievements of the public health community.

The two articles posted today come from Gregory Koblentz at George Mason University, who argues that this work was poorly justified on two fronts, scientifically and commercially (2), and from Diane DiEuliis and Gigi Gronvall from National Defense University and the Center for Health Security at Johns Hopkins University Bloomberg School of Public Health, respectively, who discuss this study in the larger context of how the risks and benefits of dual use research are assessed and managed (3). (*mSphere* asked the leadership of Tonix to submit a manuscript, but we received no response.)

It is our intent at *mSphere* to publish similar pairs of articles on controversial and cutting edge topics in the future. We are keeping our eyes open for such opportunities and welcome your ideas for potential areas that should be addressed.

These are important discussions to have, not just among those of us who pay daily attention to biosafety and biosecurity, but among the broader scientific community as a whole and especially the microbial science community. I think you will find these articles to be both thoughtful and thought-provoking.
